# A11 ISOLATION OF NON-POLAR METABOLITES IN EXCRETORY/SECRETORY PRODUCTS FROM PARASITIC HELMINTHS AND THEIR POTENTIAL AS IMMUNOTHERAPY IN INFLAMMATORY BOWEL DISEASE

**DOI:** 10.1093/jcag/gwab049.010

**Published:** 2022-02-21

**Authors:** E A Siciliani, L Leroux, M Tam, T Arai, J F Urban, R J Martin, T G Geary, M Stevenson, F Lopes, A Jardim

**Affiliations:** 1 McGill University Institute of Parasitology, Sainte Anne de Bellevue, QC, Canada; 2 Institut national de la recherche scientifique, Laval, QC, Canada; 3 McGill University Department of Microbiology & Immunology, Montreal, QC, Canada; 4 Iowa State University College of Veterinary Medicine, Ames, IA; 5 US Department of Agriculture, Washington

## Abstract

**Background:**

Parasitic helminths are known to modulate host immune responses. This is thought to be mediated by their secretome. We are interested in the excretory/secretory products and mechanisms for modulating immune dysfunction in autoinflammatory diseases.

**Aims:**

This research studies the mechanisms of immune modulation by parasitic helminths in the context of IBD. We aim to describe immunomodulatory helminth-derived metabolites (ESM).

**Methods:**

Helminth-conditioned media was used to isolate ESM, which were further purified using column chromatography.

Bone marrow (BM) derived macrophages (BMDM) from C57BL6 mice, were treated with ESP fractions from *Trichuris suis*, *Ascaris suum*, *Heligmosomoides polygyrus bakeri* or *Dirofilaria immitis*, stimulated with LPS, and secreted cytokine levels measured. Moreover, BM was cultured with or without ESM throughout differentiation to BMDM.

Colitic mice (3% DSS, 5 days) were treated with *A. suum* ESM or PBS once daily IP. Colon lengths and TNFα mRNA were measured, and histological preparations were scored to assess pathology.

Bioactive *D. immitis* ESM were fractionated using preparatory HPLC and assayed for bioactivity. Active fractions were analysed using MS/MS and fragmentation patterns and molecular weights were obtained. The active fractions are currently being studied by NMR to deduce a structure of an active metabolite.

**Results:**

BMDM treated with crude ESM decreased TNFα secretion and increased IL-10. BMDM precursors which were treated with *A. suum* ESM throughout differentiation had reduced proliferation in a dose dependent manner. These BMDM showed remodeling of BMDM metabolic pathways. Intracellular ROS production was inversely proportional to Alamar blue oxidation.

We found that ESM from *A. suum* improved DSS-colitis. Specifically, mice with DSS-induced colitis given IP ESM had longer colons, lower histolopathology score, and lower TNFα mRNA expression in gut tissue.

HPLC-fractionated *D. immitis* ESM used to treat BMDM yielded varying suppression of TNFα with LPS stimulation. MS/MS of TNFα suppressive fractions contained masses with fragmentation patterns which were detected in fractions of several of the above-mentioned parasite species. Preliminary NMR studies will determine if this represents a conserved structure.

**Conclusions:**

Helminth-derived components can immunologically polarize a response *in vitro*, as well as favour recovery in DSS colitis. Through multiple purification steps, a nearly pure fraction is found to have bioactivity, suggesting a single, bioactive molecule that is conserved across several parasitic helminths. These data are important in understanding the host-parasite interaction modulated by ESM, as well as provide therapeutic potential in IBD.

**Funding Agencies:**

NSERC, FRQNT

